# Ultra-Sensitive 3D Lateral Flow Assay Device for SARS-CoV‑2 Detection Based on One-Step Dual-Signal Amplification

**DOI:** 10.1021/acs.analchem.5c03575

**Published:** 2025-11-19

**Authors:** Yi-Ru Chiou, Wei Wang, Yuh-Shiuan Chien, Cheng-Yang Tung, Wang-Huei Sheng, Chien-Fu Chen

**Affiliations:** † Institute of Applied Mechanics, 33561 National Taiwan University, Taipei 106, Taiwan; ‡ Division of Infectious Diseases, Department of Internal Medicine, 38006 National Taiwan University Hospital, Taipei 106, Taiwan; § Center for Semiconductor Processing and Systems Research, Graduate School of Advanced Technology, National Taiwan University, Taipei 106, Taiwan

## Abstract

A one-step nucleic acid lateral flow assay (NALFA) platform was developed for on-site detection of severe acute respiratory syndrome Coronavirus 2 (SARS-CoV-2), in which no additional operation step is required for signal amplification. Polyadenine (polyA) serves as an effective anchoring block for preferential binding with the gold nanoparticles (AuNPs) surface to form probes with a high density of DNA attachment. Furthermore, bimetallic deposition, which is Au deposition and Ag staining through subsequent reductions of Au^3+^ to Au^0^ and Ag^+^ to Ag^0^ to deposit on gold nanoparticles, was used to achieve dual-signal amplification to improve colorimetric detection and sensitivity. These amplification chemistries predried on three-dimensional flow channels were constructed by stacking the papers with well-defined hydrophobic/hydrophilic areas to eliminate interference between chemistries and enhance the stability of reagents. Upon rehydration and mixing with the sample, the probes initiated a two-step metallic deposition, enabling real-time visual detection with a 2.24 nM limit, achieving 100-fold higher signal amplification than conventional LFA. Finally, the NALFA platform was successfully used to detect viral samples of the SARS-CoV-2 Omicron BA.1 variant, delivering visual results in 25 min. We expect that the developed device holds great potential in resource-limited areas.

## Graphical abstract

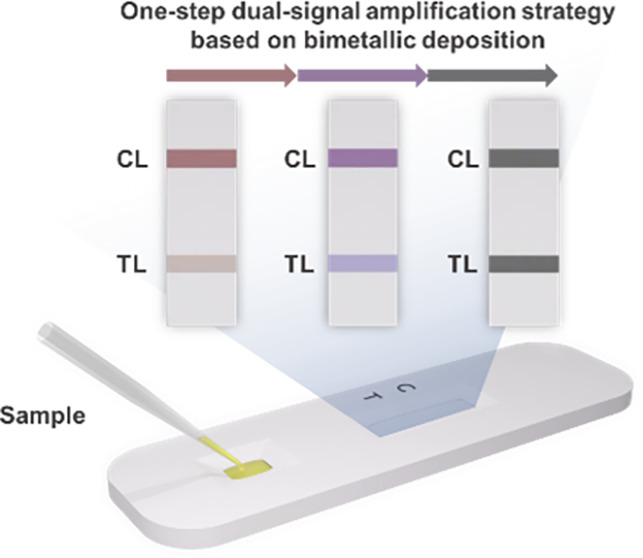

The advent of mutant viruses that may prove to be more deadly than the coronavirus in the near future has prompted the World Health Organization (WHO) to issue an alarm on the impending possibility of a worldwide pandemic. The healthcare systems worldwide are under immense strain, necessitating urgent action and preparation to ensure the availability of sufficient manpower, resources, real-time diagnostic tools, vaccination facilities, and measures to mitigate mortality risks.^1–4^ As an example, Coronavirus disease of 2019 (COVID-19), also known as SARS-CoV-2, was classified by the WHO as a global pandemic in 2020.^5^ As of June 20, 2024, the WHO figures showed over 770 million confirmed cases and more than 7 million deaths worldwide.^6^

The virus is spread through respiratory droplets, aerosols, and contact with abiotic surfaces.^7–9^ SARS-CoV-2 can induce symptoms comparable to infectious illnesses, such as fever, coughing, diarrhea, dyspnea, and loss of taste or smell.^10–12^ It can also lead to catastrophic consequences such as shock and respiratory failure, or even death.^13,14^ Despite being asymptomatic or mildly symptomatic, individuals can still transmit the virus.^15^ Unfortunately, multiple variants of SARS-CoV-2 have emerged, increasing its transmissibility and causing a rapid spike in infections.^16–18^ These situations have had a significant impact on healthcare expenditures and economic losses worldwide, straining resources and capacity.

As of now, SARS-CoV-2 is mainly diagnosed using reverse transcription polymerase chain reaction (RT-PCR).^19,20^ Due to high specificity and sensitivity, nucleic acid amplification techniques are continuously developed, such as reverse transcription loop-mediated isothermal amplification (RT-LAMP),^21^ reverse transcription recombinase polymerase amplification (RT-RPA),^22^ and clustered regularly interspaced short palindromic repeat (CRISPR).^23^ Despite advancements in SARS-CoV-2 research and technology using RT-PCR, the detection process still requires gel electrophoresis and sophisticated fluorescence instrumentation for the result analysis. In addition, the complexity, time-consuming process, and cost of requiring special equipment and skilled personnel limit the accessibility and practicality of testing.

To achieve low cost, easy operation, rapid detection, and visually observable test results, nanomaterials are now widely used for SARS-CoV-2 detection due to their unique physicochemical and optical properties.^24–26^ Colorimetric methods enable rapid and efficient detection through color changes caused by nanoparticle aggregation or metallic deposition, offering an affordable, straightforward, and rapid approach. However, these traditional methods require aqueous solutions with substantial amounts and varieties of chemical reagents, restricting their portability. Furthermore, precise control of temperature^27^ or pH levels during detection is crucial to ensure accuracy.^28^ Fortunately, LFAs have been developed to eliminate operational complexity and improve the portability for pathogen detection. AuNPs are commonly used in LFAs due to their high stability, low toxicity, and ease of control over surface modification and functionalization, as well as adjusting the particle size to generate different detection signals.^29,30^

While most LFAs use antibodies as molecular diagnostic reagents, they may suffer from cross-reactivity due to binding to similar epitopes, which can result in nonspecific detection.^31^ Although the LFA has the advantages of portability, rapid testing, and easy interpretation of test results,^3^ its narrow dynamic range can lead to poor sensitivity and result in false negatives or false positives, which may cause misinterpretation of test results.^32^ Therefore, an innovative technique for detecting LFA is necessary to increase its sensitivity and precision. To enhance LFA detection sensitivity, previous studies have explored various techniques, including colorimetry,^33^ fluorescence,^34^ electrochemistry,^35^ magnetism,^36^ surface-enhanced Raman scattering,^37^ and luminescence.^38,39^ Currently, colorimetry remains the predominant detection method in LFAs due to its ability to interpret results visually without expensive equipment, and the color changes enable rapid and on-site detection within a short time frame. In previous studies, blue-colored iridium oxide nanoparticles (IrO_2_NPs) were synthesized using sodium citrate and potassium hexachloroiridate for colorimetric detection.^40^ However, transition metals are expensive, rare, and unavailable. Previous studies have also utilized Na_2_PtCl_6_ and ascorbic acid to reduce Pt^4+^ to Pt^0^, depositing Pt on the surface of AuNP probes. With Pt on the Au@Pt NPs, catalytic activity is enabled while retaining the plasmonic properties of the original AuNPs.^41^ Following the catalytic reaction between Pt^0^ and the 3,3′5,5′-tetramethylbenzidine (TMB) reagent containing hydrogen peroxide (H_2_O_2_), the signal of LFA is amplified by the addition of an extra reagent. However, this method requires expensive reagents and more complex procedures. Therefore, to enable user-friendly detection, a copper deposition technique was employed for signal amplification. However, through reducing copper sulfate, cuprous ions were deposited onto the surface of AuNP probes maintained at a specific temperature,^42^ making practical application difficult.

To improve portability and streamline the LFA procedure, a two-dimensional paper network format for LFA detection was developed.^43^ In this approach, the HAuCl_4_ reagent and other chemical solutions were first dried on a paper. Upon rehydrating the paper and manually folding the 2D format, the AuNP probes act as catalysts, reducing Au^3+^ to Au^0^ and enabling Au^0^ to deposit simultaneously on the AuNP probe surface. Due to gold atom deposition, the AuNP probe sizes increased, resulting in signal amplification. While the method effectively reduces the detection time and operational steps, the folding design is subject to human variation. As a result, users experienced inconvenience and encountered inconsistent test results due to personal errors, thereby impeding diagnostic performance and point-of-care functionality. In order to detect infections in their early stages, LFA systems that are user-friendly, extremely sensitive, and in real-time should be created.

In this study, we developed a one-step dual-signal amplification of the NALFA platform for SARS-CoV-2 detection using a bimetallic deposition technique. The signal-amplified reagents were dried onto glass-fiber papers and stacked to produce a three-dimensional flow channel, which allowed sequential transmission of the reagents and achieved one-step rapid and sensitive detection. In addition to reducing human errors and reagent consumption, this approach offers advantages such as portability and user-friendly detection. We utilized gold nanoparticles as probes due to their high stability and excellent colorimetric capability. The polyA structure at the end of the oligonucleotides anchors to AuNPs surfaces through strong adenine–gold affinity, forming dense and orderly monolayers that not only ensure stable binding but also shield the bare gold surface and minimize nonspecific interactions.^44^ Then, through salt-aging methods, we efficiently synthesized AuNP-based colorimetric probes with an upright conformation.^45^ Compared to previous thiol-functionalization, our approach not only increased hybridization efficiency but also reduced nonspecific adsorption.^46^ To enhance the sensitivity of the NALFA platform, we demonstrated a sequential gold and silver deposition technique on AuNP probes to amplify the signal lines. Various chemistries were predried on glass-fiber papers to facilitate precise control and minimize reagent volume, enhancing portability and ease of storage. Through a three-dimensional flow channel created by stacking components, the amplification chemistries were separated exactly and sequentially transmitted. The first amplification was achieved by depositing gold atoms onto the surface of the AuNP probes through reduction of chloroauric acid (HAuCl_4_) to Au^0^ by reducing agents. In the stacking of the paper layers, a time difference allowed for the sequential reduction of Ag^+^ from AgNO_3_ to Ag^0^ with hydroquinone,^47^ which then deposited silver atoms onto the AuNPs surfaces. The LFA signals were effectively amplified due to the dual-metal deposition technique, which greatly improved the detection sensitivity. Finally, we successfully applied the one-step dual-signal-amplified NALFA platform to detect SARS-CoV-2 in clinical samples, achieving visually interpretable results within 25 min without the need for reagent addition or complex operations. The platform eliminates manual steps and external reagents, using dry-stabilized components embedded in a 3D paper-based system suitable for room-temperature storage. It also supports rapid probe customization, making it ideal for point-of-care diagnostics, mass screening, and detection of emerging pathogens. Notably, the high sensitivity of this method is especially valuable for detecting infections with low viral loads, such as those in asymptomatic or presymptomatic individuals who are often responsible for silent transmission. Early detection in such cases facilitates timely isolation, limits viral spread, and allows prompt medical intervention—crucial during outbreaks or among vulnerable populations.

## One-Step Dual-Signal Amplification Strategies for the NALFA

We developed a one-step dual-signal amplification NALFA platform for SARS-CoV-2 E gene detection (Video S1). This platform is composed of polyA-mediated formation of DNA-AuNPs and a three-dimensional flow pathway channel created by paper stacking. The detection process was simplified to a one-step operation and achieved dual-signal amplification, as shown in Figure 1. The NALFA platform consists of standard lateral flow paper components: sample pads, conjugate pads, nitrocellulose membrane, and absorbent pads. It also includes functional elements for signal amplification, including wax pads, amplification glass-fiber papers, and cellulose papers. Figure 1A provides a visual representation of the components. Cellulose papers were used to facilitate the fluidic channels, while the wax pads controlled the liquid flow direction and prevented interference between different amplified reagents. Importantly, precise temporal control of the amplification was achieved through sequential fluidic delivery. If amplification reagents were preimmobilized on the strip, they could interact with the sample matrix or ambient moisture prior to target binding, causing undesired premature reactions and elevated background signals. By contrast, introducing amplification reagents only after the target had been captured ensured that amplification was strictly coupled to the analyte presence, thereby enhancing specificity and reliability.

**Figure 1:**
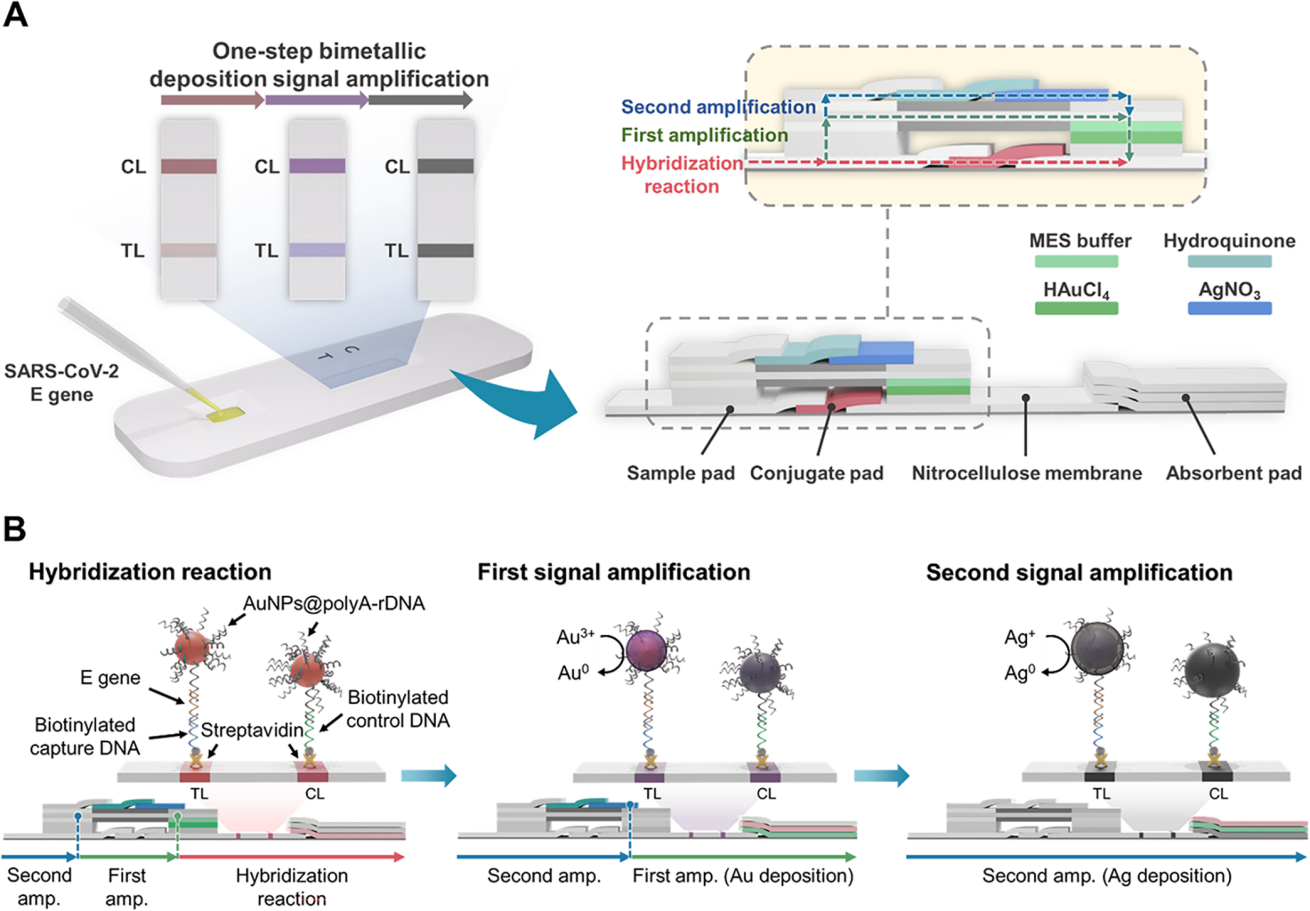
(A) Schematic illustration of the NALFA platform for SARS-CoV-2 detection using one-step bimetallic deposition for dual-signal amplification. (B) Mechanism of bimetallic-deposition-based signal amplification and detection on the NALFA platform.

On the other hand, the stacked papers were treated with signal amplification reagents such as HAuCl_4_, 2-(*N*-morpholino)­ethanesulfonic acid (MES buffer), AgNO_3_, and hydroquinone and then arranged in a particular order to create separate flow paths. This design enables precise control of fluids during detection, with hybridization reactions and signal amplification occurring sequentially. Streptavidin has a high binding affinity for biotin,^48^ and therefore, biotinylated capture DNA and biotinylated control DNA were securely attached to the test line (TL) and control line (CL), respectively, in our modified biotin–streptavidin strip. Additionally, the surfaces of AuNPs were modified with polyA, which serves as a specific recognition sequence (Table S1). The polyA adsorbed on the surface of AuNPs with high affinity and produced an upright conformation, improving the efficiency of hybridization.^49^ Samples (SARS-CoV-2 E-gene) were efficiently hybridized to the detector probes (polyA DNA-AuNPs) and accumulated on the TL and CL or flowed through the nitrocellulose (NC) membrane. Visual verification of the distinct red signals was possible due to surface plasmon resonance (SPR) effects. To achieve successive signal amplification, stacked paper layers were utilized to produce fluid transmission. HAuCl_4_ and MES buffers were predried on the glass-fiber papers and flowed over the TL and CL where polyA DNA-AuNPs were captured, resulting in the reduction of Au^3+^ to Au^0^ and deposition on AuNPs. In the signal results, the deposition leads to a color change from red to purple owing to the increasing size of the AuNPs. Subsequently, silver atoms were reduced from predried papers of AgNO_3_ and hydroquinone, achieving secondary signal amplification due to the reduction of Ag^+^ from AgNO_3_ to Ag^0^, and a color change from purple to black (Figure 1B). Importantly, the detection process is a one-step operation, eliminating complicated manipulations or the addition of supplementary reagents to amplify the detection signals and increase the sensitivity.

## Synthesis and Characterization of the polyA DNA-AuNPs

Poly A-mediated formation of DNA-AuNPs were synthesized as described in a previous work.^50^ Briefly, the polyA tail was anchored on functional oligonucleotides on the surface of AuNPs followed by the addition of sodium chloride for salt-aging to accelerate the reaction. After incubation for 24 h at 4 °C and purification by centrifugation, polyA DNA-AuNPs were formed (Figure 2A). The UV–vis absorption spectra of citrate-synthesized AuNPs showed a characteristic surface plasmon resonance (SPR) peak at 518 nm. Upon hybridization with polyA-tailed DNA, the formation of polyA-DNA-AuNPs led to a redshift in the SPR peak to 526 nm (Figure 2B), indicating successful surface modification. Notably, a distinct absorption peak at 260 nm, corresponding to the nucleobase-rich DNA layer, was also clearly observed in the polyA DNA-AuNPs spectrum. In contrast, the citrate-AuNPs exhibited no such peak at 260 nm, further confirming the presence of DNA exclusively in the functionalized nanoconjugates.^51,52^ In addition, the particle size was found to increase, as measured by dynamic light scattering (DLS); citrate-stabilized AuNPs and polyA DNA-AuNPs showed a hydrodynamic diameter of 12 and 23 nm, respectively (Figure 2C).^53,54^ The zeta potential values indicated that the surface charge decreased slightly as a result of adsorption of polyA onto the surface of the AuNPs compared with bare AuNPs from −42.7 to −50.7 mV (Figure 2D),^55^ and all these results confirmed the formation of polyA DNA-AuNPs. Subsequently, polyA DNA-AuNPs as probes were used in NALFA testing, and the synthesis conditions, concentration, and incubation time of streptavidin sprayed onto TL and CL of the NC membrane were optimized. The maximum signal intensities were observed with activated AuNPs incubated with polyA DNA for extended periods of more than 24 h (Figure S1A). Additionally, the streptavidin–biotin interaction is the most common method for immobilizing streptavidin on solid surfaces as there is a significant and strong noncovalent interaction between streptavidin and biotin, and the dissociation constant of around 1 × 10^–14^ M for biotin binding to streptavidin based on previous studies.^56,57^ Further analysis determined the concentration of streptavidin immobilized on the TL and CL. Using a 1:4 molar ratio of streptavidin to biotinylated DNA, we immobilized different concentrations of streptavidin on the TL and CL for testing. Significant signals on the TL and CL were observed using streptavidin concentrations of 2.0 and 1.0 mg/mL, respectively (Figure S1B,C). Finally, the NALFA platform under optimized conditions and all images were recorded at different times using ImageJ software for analysis, as shown in Figure S2A.

**Figure 2:**
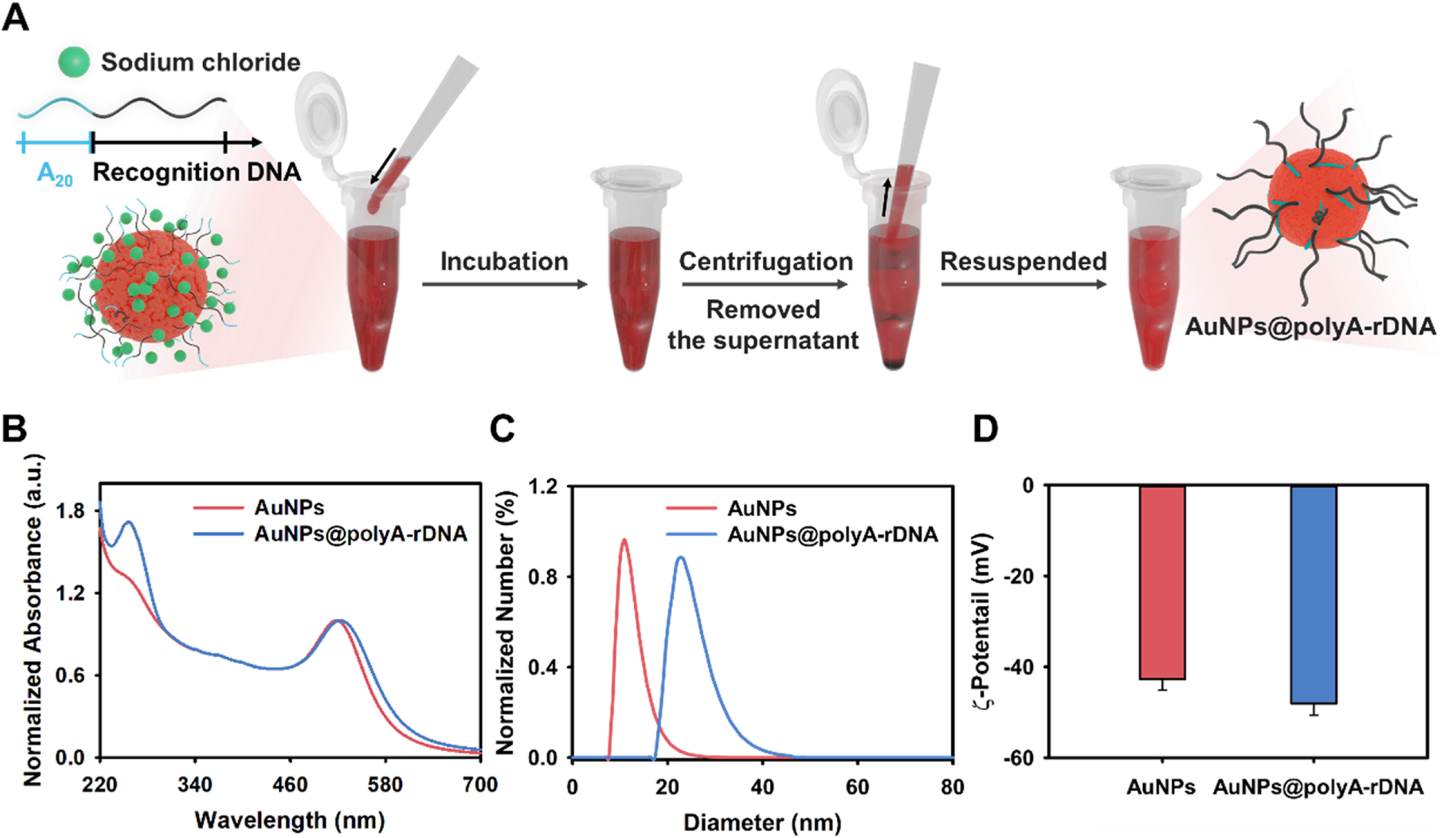
(A) Schematic diagram of the synthesis of polyA DNA-AuNPs conjugate. (B) UV–vis absorption spectra, (C) DLS, and (D) zeta potential of AuNPs and polyA DNA-AuNPs, respectively.

## Amplification Chemistries Optimization

To overcome the previous limitations of LFA sensitivity, it was necessary to develop Au deposition and Ag staining signal amplification in one step, which depended on particle size and metal deposition to observe the changes in color of lines on the LFA as shown in Figure 3A. The initial signal amplification through a gold deposition technique involved the reduction of Au^3+^ ions from HAuCl_4_ to Au^0^ followed by using an MES buffer. Gold atoms were chemically reduced and deposited on polyA DNA-AuNP probes. With the increase in size of the probes due to metal deposition, the surface plasmon resonance of the gold nanoparticles triggered color transition from pink to purple.^58^ Subsequently, the reduction of Ag^+^ from AgNO_3_ to Ag^0^ using hydroquinone initiated silver staining,^59^ resulting in a strong color change from purple to black, achieving the second round of signal amplification. To optimize the dosage of amplification chemistries and ensure the metal deposition strategy applied to the LFA platform, the concentrations of HAuCl_4_ (concentrations of 0.5, 1.0, 2.0, 2.5, and 5.0 mM) on glass-fiber papers for gold deposition were fine-tuned. The TL and CL signal strength increased with increasing concentrations of HAuCl_4_, and the signal plateaued at about 2.5 mM as observed in Figure 3B. Moreover, the pH of the MES buffer solution was controlled at pH 4.0, 5.0, 6.0, and 7.0 to determine the effect of pH on signal enhancement.^42^ The results demonstrated that as the pH value increased to neutral or a pH value below 4, the background signal of the NALFA also increased, leading to a decrease in the signal-to-noise ratio and accuracy of visual assessment (Figure 3C). For accurate visual interpretation of the CL and TL signals, an MES buffer with an optimal pH 5.0 was selected to achieve a higher signal-to-noise ratio (SNR). Following the determination of the optimal pH condition, various concentrations of MES buffer (conc. 5, 10, 50, 100, and 150 mM) were dried on the glass-fiber papers. In the presence of MES buffer concentrations between 50 and 150 mM, finally, TL signal strengths remained constant since HAuCl_4_ reacted completely with the MES buffer in the TL before passing through the CL (Figure 3D). For follow-up tests, we finally used 50 mM MES buffer. Further, the silver staining technique was carried out for drying different concentrations of AgNO_3_ solution (0.03%, 0.15%, 0.3%, and 3%; w/w) and hydroquinone solution (0.3%, 1.5%, 3%, and 6%; w/w) on glass-fiber papers for signal enhancement (Figure 3E,F). When 0.3% AgNO_3_ and 3% hydroquinone were used as amplification reagents, satisfactory results were observed for visual interpretation and software analysis. In addition, images of the NALFA platform were observed and obtained through a scanning electron microscope to realize the signal amplification process. The average particle size on the TL of NALFA without signal amplification was about 15.2 ± 1.3 nm (Figure 4A). After Au deposition amplification, the average particle size increased to approximately 42.8 ± 9.7 nm, indicating that gold atoms can be stably deposited on the surface of polyA DNA-AuNP probes (Figure 4B). Dual-signal amplification by combining Au deposition with Ag staining using the NALFA platform resulted in an increase in the particle size to about 93.1 ± 7.6 nm as shown in Figure 4C. As further confirmed by Figure S3 the size variation and the success of signal amplification were validated. Observation and detection sensitivity of the NALFA platform were enhanced due to the obvious color and particle size changes after metal deposition. Observation and detection sensitivity of the NALFA platform were enhanced due to the obvious color and particle size change after metal deposition.

**Figure 3:**
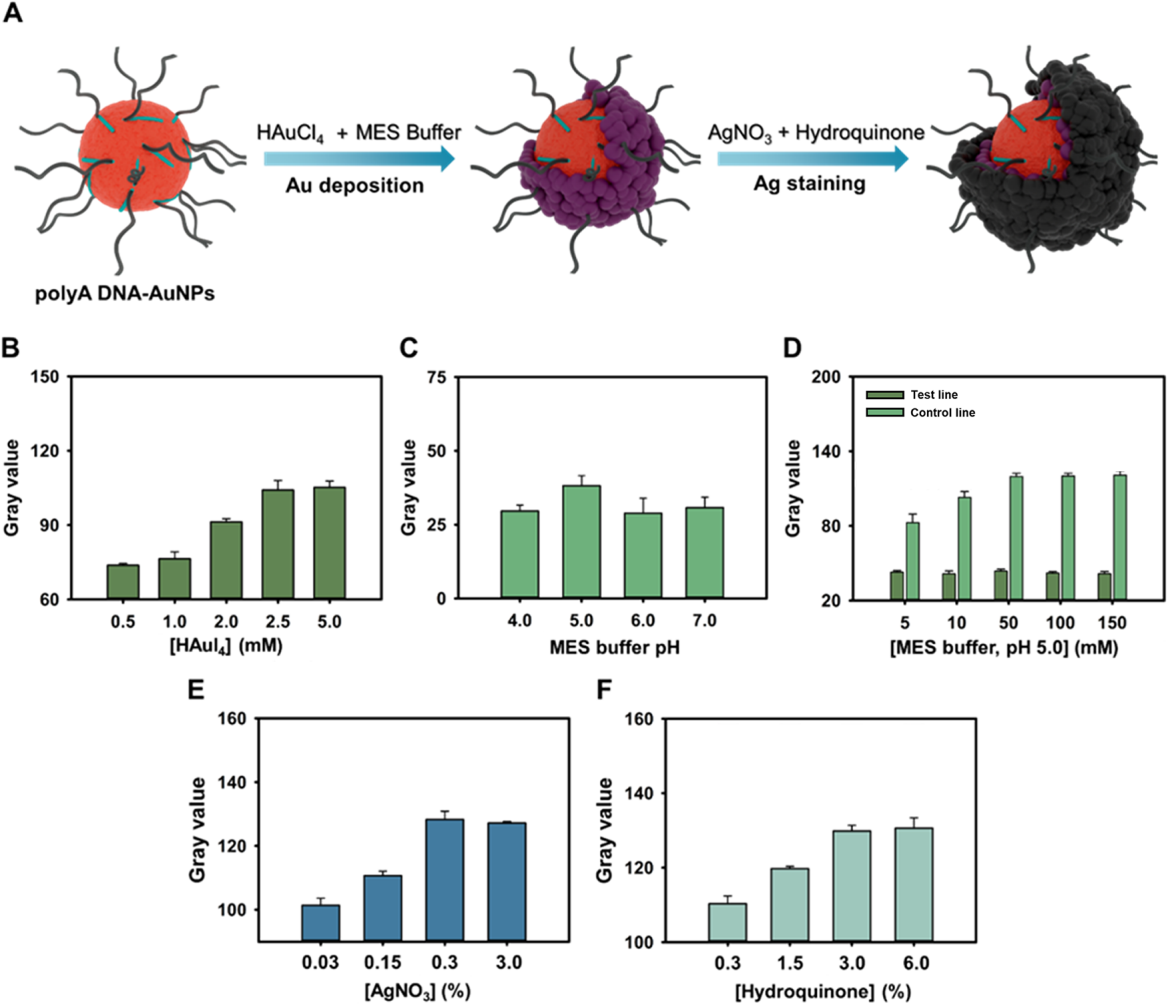
(A) Schematic of bimetallic deposition utilizing amplified chemistries for dual-signal amplification with AuNPs@polyA-rDNA probes on the NALFA platform. Exploration of (B) various HAuCl_4_ concentrations and (C) pH values of MES buffer drying on storage pads for the first amplification. (D) Evaluation of MES buffer solution at pH 5.0 at different concentrations for visual detection. Different concentrations of (E) AgNO_3_ and (F) hydroquinone dried on storage strips were used for the second deposition. All of the imaging data were collected and analyzed using grayscale values of ImageJ software (*n* = 3).

**Figure 4:**
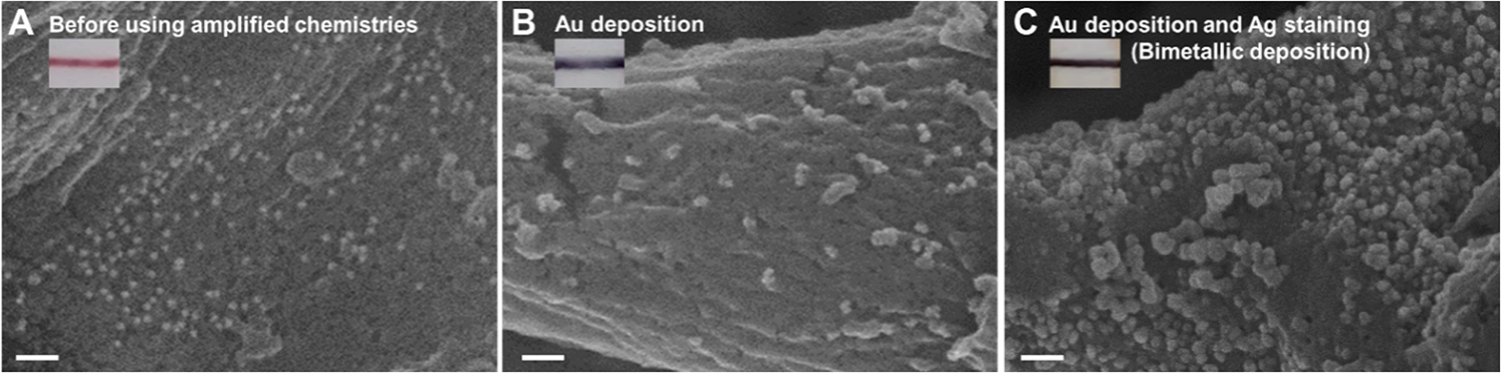
SEM images of polyA DNA-AuNP probes on TL images from NALFA platforms at different stages of results. (A) Standard method without using amplification chemistries. (B) Amplification chemistries of Au deposition were used. (C) Au deposition and Ag staining were utilized for dual-signal amplification methods. Scale bars: 100 nm.

## NALFA Platform Design with Stacked Structures for Time-Dependent Sequential Signal Amplification

The NALFA platform was improved by combining optimized conditions of amplification chemistries and fluidic channels by using paper stacking techniques to achieve one-step dual-signal amplification. First, the amplification chemistries were predried on Drying amplification chemistries on paper can enhance their storage time and stability of reagents. Dry storage not only simplifies reagent packs and uses, but also prevents reagents from severe decaying and reduces their sensitivity to environmental factors, such as hydration reaction, pH variation, and light-induced degradation.^60,61^ Figure 5A shows that Au deposition amplification chemistries and Ag staining amplification chemistries were dried on glass-fiber papers. Wax papers were used as a hydrophobic interlayer to avoid interference of reagents or amplification. A commercially available wax printer was used to create the wax image on the paper surface. After that, the papers were heated to a relatively low temperature (*T* ≈ 110 °C) to melt the wax and the driving force that moved the molten wax through the paper was capillary action.^62^ These well-formed hydrophobic sheets were employed as hydrophobic channel walls, which effectively and completely enclosed the channels from the exterior environment, decreasing contamination risks and significantly lowering reagent interference.^63^ With a 3D-printed cartridge, multilayered papers can also be fixed and stacked precisely, enabling rapid detection and portability.

**Figure 5:**
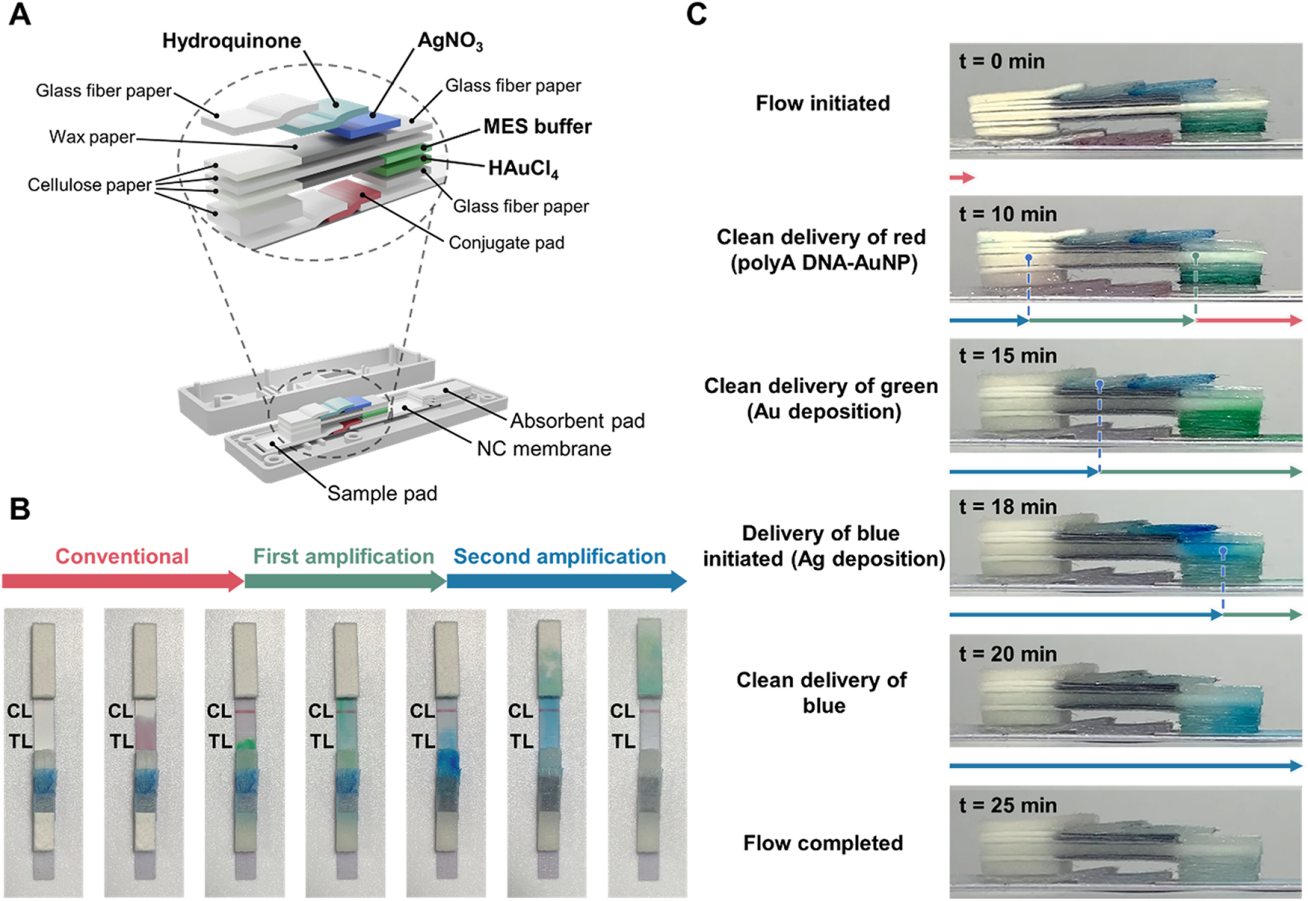
(A) Schematic diagram of the stacked structure of the amplification parts and components in the NALFA platform. For proof-of-concept and time-dependent transmission by visible pigments, (B) a top view and (C) a side view of the NALFA platform.glass-fiber papers. Then, liquids were sequentially transmitted to the detection zones through fabricating papers with hydrophilic and hydrophobic properties to achieve dual-signal amplification.

To confirm the paper stacking and visualize the detection process, the amplification chemistries of Au deposition and Ag staining were represented by green and blue pigments, respectively. Thus, polyA DNA-AuNP probes, green pigments, and blue pigments were predried on the papers, and the process flows were recorded from a top and side view (Figure 5B,C). As shown in Figure 5C, the hybridization reaction of polyA DNA-AuNP probes was completed in approximately ten min, initiating the release of gold deposition amplification chemistries. Around 15 min later, the amplification chemistries for Au deposition started, followed by the flow of Ag staining amplification chemistries at about 18 min. In the 20 min interval, the amplification chemistries for Ag staining reached the detection zones. We analyzed the process at different times to ensure adequate reaction of the amplification chemistries, and Figure S3 demonstrated that the amplified reaction was completed within 25 min. By using only one step of operation, this approach successfully enhanced the sensitivity of the NALFA platform through a subsequent amplification strategy.

## Sensitivity and Comparison of Colorimetric Amplification Methods for the NALFA

We further evaluated the sensitivity of the NALFA platform using different amplification chemistries for signal amplification after undergoing hybridization and colorimetric changes in TL and CL, respectively. Figure 6A presents the linear calibration curve for the diagnostics of SARS-CoV-2 E gene standards at concentrations ranging from 10 to 1000 nM without the use of amplification chemistries with a limit of detection (LOD) of 310 nM (*R* ^2^ = 0.99, *n* = 5). Under Au amplification, the results showed a calibration curve with a LOD of 24.4 nM (*R* ^2^ = 0.98, *n* = 5) that reached approximately 10-fold enhancement in the signal response (Figure 6B). Finally, through the dual-signal amplification technique of Au deposition and Ag staining, LOD further decreased to 2.24 nM as determined by the linear calibration line (*R* ^2^ = 0.99, *n* = 5) in Figure 6C. Data were collected in duplicate for all experiments. Using ImageJ Software, the calibration curves were generated and the LODs were calculated by using the formula (LOD = 3.3 × SD_blank/slope_),^64^ where SDblank represents the standard deviation of blank measurements, and slope refers to the slope of the linear regression obtained from the calibration curve. Each condition was tested at least five times independently to ensure reproducibility. The detection sensitivity increased up to 138-fold compared with the conventional LFA, and the dark signal results can be easily interpreted through visual observation at the same standard concentration. In addition, to validate the advantages of a dual-signal amplification strategy based on bimetallic deposition, we employed the previously mentioned copper deposition approach for signal enhancement. By copper deposition, the LOD obtained from the calibration curve was 40 nM (*R* ^2^ = 0.99, *n* = 5). For the same concentration of the SARS-CoV-2 E gene, the signal intensities using a dual-signal amplification strategy were superior to those obtained with copper-deposition-assisted signal amplification (Figures S4 and S5).

**Figure 6:**
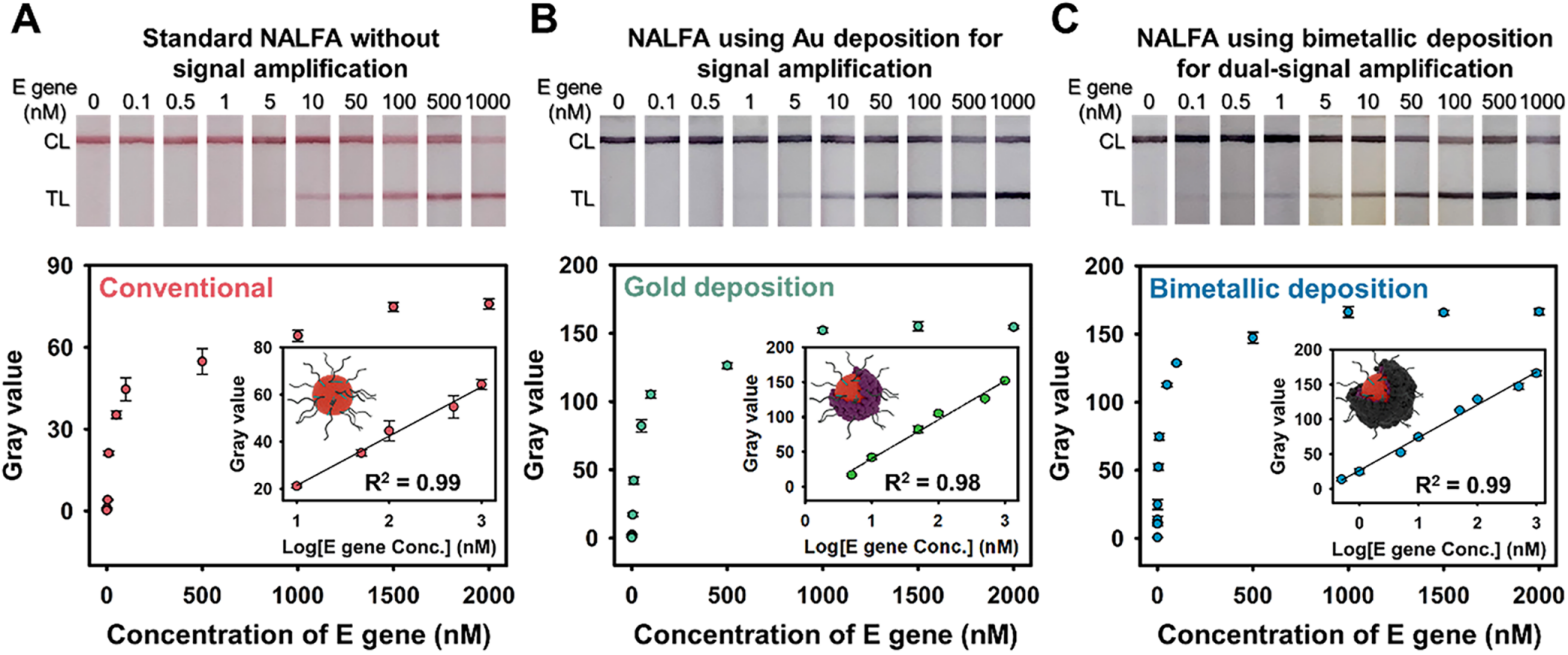
(A) Colorimetric intensities of test lines obtained on the NALFA platform for different deposition techniques and SARS-CoV-2 E gene standard concentrations. (A) Standard NALFA without signal amplification, (B) after Au deposition technique, and (C) after Au deposition and Ag staining as bimetallic deposition techniques for dual-signal amplification. The grayscale values of all imaging data were estimated using ImageJ software to obtain the calibration curve.

## Detection of Virus Sample

Clinical samples taken from critically ill patients at the National Taiwan University Hospital were used to evaluate the viability of the dual-signal amplification approach using bimetallic deposition on the NALFA platform. The actual samples and established concentrations of SARS-CoV-2 (Omicron BA.1) were treated in a biosafety level-3 laboratory (BSL-3 Lab).

As shown in Figure 7A, different concentrations of BA.1 (3.0 × 10^8^, 6.0 × 10^8^, 6.0 × 10^9^, 6.0 × 10^9^, 3.0 × 10^10^, 6.0 × 10^10^, 3.0 × 10^11^, and 6.0 × 10^11^ copies/μL) were added to the NALFA platform for testing. As the polyA-DNA-modified AuNPs complexes migrate to test and control zones, hybridization occurs, followed by gold deposition and silver staining. The colorimetric signals on the NALFA platform were recorded using a smartphone and analyzed through ImageJ software to obtain gray value results. Figure 7B demonstrates a strong correlation between signal intensity in the detection of BA.1 virus samples and standards. A linear relationship (*R* ^2^ = 0.99) was observed between the gray value and logarithmic BA.1 concentration in the range from 3.0 × 10^8^ to 6.0 × 10^11^ copies/μL, indicating the combination of the NALFA platform and dual-metal deposition amplification method has great potential for practical applications in detecting highly infectious or mutated viruses. Moreover, as summarized in Table S2, the recovery rates calculated by comparing the detected concentrations with the original spiked values across the same concentration range revealed a high analytical accuracy and reproducibility. The recovery values ranged from 95.4% to 111%, with most values falling within the 98–109% interval, further supporting the reliability and robustness of our detection system across diverse viral loads.

**Figure 7:**
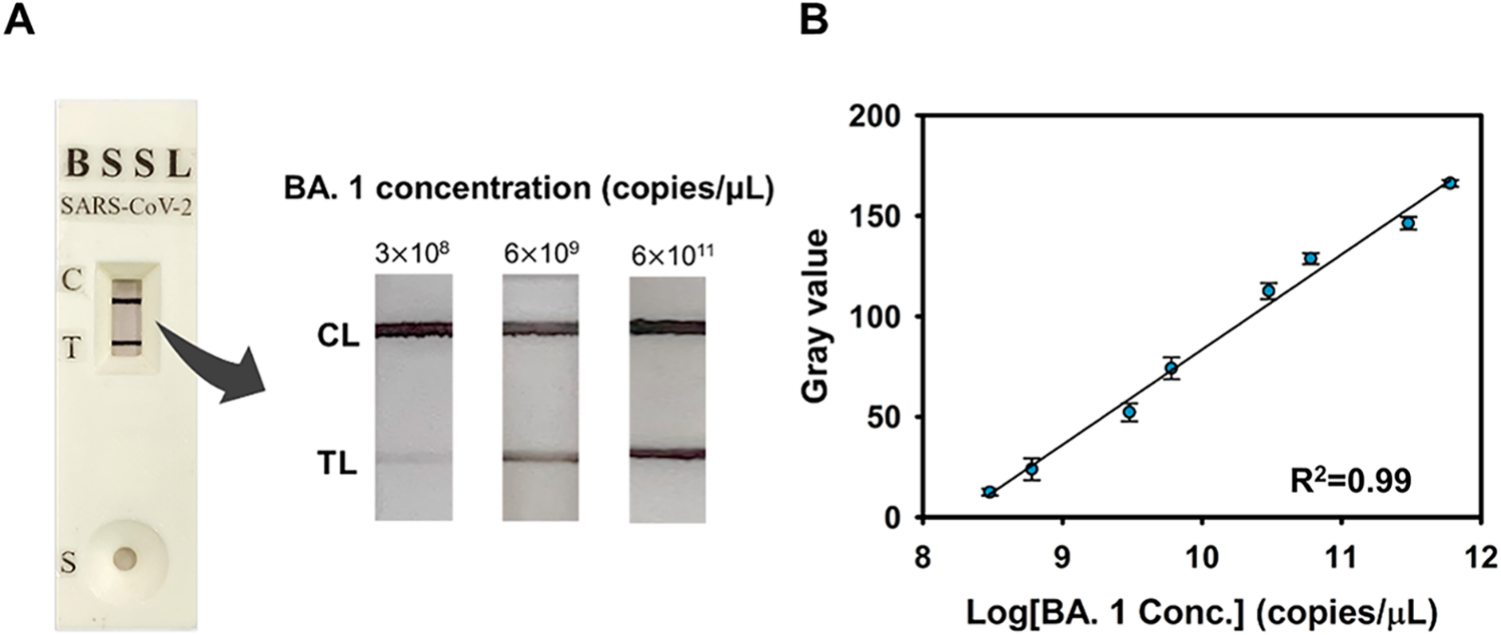
Detection of the Omicron BA.1 samples. (A) Test results were recorded with a smartphone using a dual-signal amplification NALFA platform. (B) Calibration curve was obtained by analyzing the TL in grayscale for each image to determine the concentration of variant samples. Data are expressed as mean ± SD of three measurements.

## Conclusions

In this study, we developed a highly sensitive, easy-to-use, and NALFA platform for on-site detection of SARS-CoV-2. PolyA DNA-AuNP probes were prepared in an upright configuration to efficiently enhance hybridization. Amplification chemistries of Au deposition and silver staining were predried on the glass-fiber papers and precisely stacked, resulting in sequential transmission for dual-signal through one-step rehydration. Drying technologies are critical to the function of paper on the microfluidic NALFA platform. Dry- stored reagents are easier to operate and have fewer packing procedures. They are less vulnerable to environmental influences, such as light-induced degradation, pH fluctuations, and hydration reactions. In order to enable the reduction of Au^3+^ to Au^0^ and Ag^+^ to Ag^0^ to deposit on gold nanoparticles for dual-signal amplification and to remove interference between chemical reagents, three-dimensional flow channels were created by stacking hydrophobic/hydrophilic sheets.

The NALFA platform enables sequential liquid transport without adding excess reagents to achieve dual-signal signal amplification, allowing the detection process to be completed within 25 min with a limit of detection 1 nM. Our methods show good competitiveness compared with other state-of-the-art detection technologies (Table S3). Moreover, it provides visible results in a short time, making this platform appropriate for application in diverse point-of-care testing. The design is also manufacturing-friendly: the multilayer assembly and bimetallic amplification are compatible with roll-to-roll production, with an estimated material cost of about USD 3 per strip, expected to decrease further upon scale-up. Overall, the dual-signal amplification strategy based on bimetallic deposition in a one-step process effectively addresses the issue of limited detection sensitivity common to conventional methods and has been successfully applied to detect SARS-CoV-2 variants. Toward the future, as viruses, variants, and a newly discovered Disease X continuously merge,^65^ DNA sequences on the probes can be modified to rapidly detect novel variants. The platform can also be extended for multiplex detection by incorporating additional DNA probes or primers. In addition, to overcome the cold-chain requirements for reagents such as streptavidin, antibody/antigen-loaded strips were vacuum-sealed with protective excipients (e.g., sucrose) and stored at 4 °C, which maintained reliable performance for at least 75 days.^66^ Moreover, excipients like sucrose and trehalose can be integrated during lyophilization to stabilize protein structures, thereby ensuring long-term storage stability at ambient temperature. Furthermore, visual detection is possible in resource-limited areas, facilitating early detection, control, and containment of viral occurrence and transmission.

## Supplementary Material





## Notes

Materials and experimental methods, supporting figures of the optimization of DNA–AuNPs and binding analysis, time-dependent amplification of the NALFA platform using the E-gene standard, characterization of the amplification structures, time-dependent copper-based signal amplification and reagent optimization on the NALFA platform, Cu deposition–based amplification, and tables of oligonucleotides sequences, recovery experiment of Omicron BA.1 samples, and comparison with other methods (PDF)
Operation process of NALFA (Video S1) (MP4)
