# Supplementary material for: Ultra-Sensitive 3D Lateral Flow Assay Device for SARS-CoV‑2 Detection Based on One-Step Dual-Signal Amplification
Source: Anal Chem. 2025 Nov 19;97(48):26429–38. doi: 10.1021/acs.analchem.5c03575 (PMC12874218; doi:10.1021/acs.analchem.5c03575)
Supplement: Supplementary file 1 [file ac5c03575_si_001.pdf]

Supporting Information

An Ultra-Sensitive 3D Lateral Flow Assay Device for SARS-CoV-2 Detection Based on One-Step Dual-Signal Amplification

Yi-Ru Chiou[a†], Wei Wang[a†], Yuh-Shiuan Chien[a†], Cheng-Yang Tung[a], Wang-Huei Sheng[b], and Chien-Fu Chen[*ac]

[a]Institute of Applied Mechanics, National Taiwan University, Taipei 106, Taiwan

[b]Division of Infectious Diseases, Department of Internal Medicine, National Taiwan University Hospital, Taipei 100, Taiwan

[c]Center for Semiconductor Processing and Systems Research, Graduate School of Advanced Technology, National Taiwan University, Taipei 106, Taiwan

*Corresponding author: Chien-Fu Chen (stevechen@ntu.edu.tw)

Content

Chemicals....S3
Instrumentation....S3
Synthesis of AuNPs and polyA DNA-AuNPs....S4
Synthesis of biotinylated DNA with streptavidin....S4
Fabrication of the NALFA platform and functional elements....S4
Preparation of Test Strip and Amplification Part....S5
Optimization of chemicals for Au deposition amplification....S5
Optimization of chemicals for Ag staining amplification....S5
Evaluation of NALFA platform sensitivity....S6
Analysis of SARS-CoV-2 Samples....S6
Table S1....S7
Figure S1....S8
Figure S2....S9
Figure S3....S10
Figure S4....S11
Figure S5....S12
Table S2....S13
Table S3....S14
REFERENCES....S15

EXPERIMENTAL SECTION

Chemicals

The SARS-CoV-2 E gene, recognition DNA, biotinylated capture DNA, biotinylated control DNA, and the forward and reverse primer of SARS-CoV-2 were provided by Protech Technology Enterprise Co., Ltd (Taipei, Taiwan). KAPA SYBR® FAST qPCR Master Mix (2×) was purchased from Kapa Biosystems (MA, USA). Nuclease-free water was purchased from Bio-Genesis Technology, Inc (Taipei, Taiwan). Phosphate buffered saline buffer (PBS, pH 7.4), sodium chloride (99%), phosphate buffered saline containing TWEEN® 20 (PBST, pH 7.4), bovine serum albumin (BSA), saline-sodium citrate buffer (SSC; 20×), 2-(N-Morpholino) ethanesulfonic acid (MES, 99%), silver nitrate (99%), hydroquinone (99%), copper sulfate pentahydrate (99%), and L-ascorbic acid were purchased from Sigma-Aldrich (MO, USA). Sodium citrate and sucrose were purchased from Avantor Performance Materials Inc (PA, USA). Tetrachloroauric acid (99%), streptavidin, and SuperScript™ III First-Strand Synthesis SuperMix were obtained from Thermo Fisher Scientific (MA, USA). The SARS-CoV-2 Omicron variant (BA.1) was obtained from National Taiwan University Hospital (Taipei, Taiwan). The paper substrates of glass fibers (SB08), polyester fibers (DL42), absorbent pads (SX27), and adhesive backing pads were purchased from Kinbio Tech (Shanghai, China). Nitrocellulose membrane (CN140) was purchased by Sartorius Co., Ltd (Göttingen, Germany).

Instrumentation

The hydrophobic barriers of cellulose substrates were patterned by the ColorQube 8570 wax printer from Xerox Corporation (Connecticut, USA). The KUBOTA 3520 micro refrigerated centrifuge was obtained from Double Eagle Enterprise (New Taipei, Taiwan). The LEGATO® 110 syringe pump was purchased from KD Scientific Inc (MA, USA). The pH value was measured using the FiveEasy Plus pH meter FP20-Micro-Kit from Mettler Toledo (Zürich, Switzerland). Deionized water was obtained using the Sartorius Arium® mini plus lab water system (Göttingen, Germany). The cartridge was manufactured using an Ultimaker® S3 3D printer (Utrecht, Netherlands). The UV–Vis absorption spectra of AuNPs and polyA DNA-AuNPs were recorded using the Jasco V-730 (Tokyo, Japan). Scanning electron microscopy images were recorded through a Hitachi S-4800 field emission SEM (Tokyo, Japan). Zeta potential and particle size were determined with an Otsuka Electronics ELSZ-2000 (Osaka, Japan).

Experimental Section

Synthesis of AuNPs and polyA DNA-AuNPs

AuNPs with a diameter of 13 nm were synthesized according to a previously reported method, [1] and the concentration was determined using Beer's law. The polyA DNA-AuNPs conjugate was prepared based on a previous report. [2] Briefly, a mixture of 10 μM polyA DNA and 13 nm AuNPs in a ratio of 8:1was incubated for 10 min at room temperature, and NaCl solution (150 mM, 100 μL) was added to the mixture and incubated at 4°C for 24 h. Subsequently, the mixture was subjected to three centrifugal washing steps to eliminate excess reagents (13200 rpm, 20 min, 4°C), and the resulting polyA DNA-AuNPs were thoroughly resuspended in 600 μL of 1× PBST (pH 7.4) containing 10% sucrose and stored at 4°C until further use.

Synthesis of biotinylated DNA with streptavidin

Biotinylated DNA was prepared using a typical conjugation procedure. [3] A mixture of 112 μL streptavidin solution (5.0 mg/mL) and 40.6 μL capture DNA solution (1.0 mM) in 127 μL of PBS (10 mM, pH 7.4) was incubated at 4°C for 1 h to obtain a streptavidin-biotinylated capture DNA solution. Similarly, streptavidin-biotinylated control DNA was synthesized using 105 μL streptavidin solution (5.0 mg/mL) and 38.2 μL control DNA solution (1.0 mM) in 350 μL of PBS (10.0 mM, pH 7.4) and followed by incubation at 4°C for 1 h to a obtain streptavidin-biotinylated control DNA solution. The resulting solution of biotinylated DNA was stored at 4°C until use.

Fabrication of the NALFA platform and functional elements

The NALFA platform (65 × 5.0 mm^2) consisting of a sample pad, a conjugate pad, an nitrocellulose (NC) membrane, and an absorbent pad were prepared using a typical process of lateral stacking and lamination onto a plastic adhesive backing pad. The sample pad was pretreated with running buffer containing 1% (w/w) BSA solution in 1× PBST (pH 7.4), dried at 37°C for 2 h, and stored at room temperature for further use. The conjugate pad was added to 40 μL polyA DNA-AuNPs solution and dried at 37°C for 1 h. The test line (TL) and control line (CL) on the NC membrane were sprayed with streptavidin-biotinylated capture DNA solution and streptavidin-biotinylated control DNA solution using the dispenser at a rate of 0.2 μL/s, respectively, and the membrane was dried at 37°C for 1 h. An absorbent pad with a length of 17 mm was used. Liquid backflow was prevented by absorbing excess reagents to reduce the background interference. Finally, the typical NALFA platform involves sequentially attaching the NC membrane, conjugate pad, sample pad, and absorbent pad to a plastic adhesive backing pad.

Preparation of Test Strip and Amplification Part

The amplification part was constructed by layer-by-layer lamination of the functional elements (Figure 4A), which included wax pads, glass-fiber papers with amplified chemicals, and cellulose pads. First, the wax pads were prepared using cellulose paper as a substrate, and hydrophobic barriers (18×5 mm^2) were made by melting printed wax into the filter paper at 110°C for 10 minutes. Also, two contact glass-fiber papers for the Au deposition amplification part were treated with 20 µL of 2.5 mM $HAuCl_4$ and 20 µL of MES buffer (150 mM, pH = 5.0), respectively. Similar to the Au deposition amplification part, two contact glass-fiber papers for Ag staining amplification were treated with 20 µL of 0.3% $AgNO_3$ and 20 µL hydroquinone (3.0%, pH = 4.0), respectively. All glass-fiber papers were dried at 37°C for 30 minutes. The cellulose pads and papers were prepared to have lengths of 9.0 mm and 25.0 mm, respectively, and each functional element was prepared at 5.0 mm width. The functional elements were vertically stacked and laminated on glass-fiber papers to produce an amplification part. In addition, SolidWorks software and a 3D printer were used to design and print out the cartridge for reliability and portability during the detection process.

Optimization of chemicals for Au deposition amplification

The TL and CL capturing polyA DNA-AuNP probes after hybridization exhibited a red signal band. Subsequently, to evaluate the stability of the reagents and concentrations used for Au deposition amplification, glass-fiber papers were treated with different concentrations of $HAuCl_4$ (0.5, 1.0, 2.0, 2.5 and 5.0 mM) and MES buffer (5, 10, 50, 100 and 150 mM) at different pH values (pH 4.0, 5.0, 6.0 and 7.0) and dried at 37 °C for 30 minutes. The optimization of $HAuCl_4$ concentration was performed using 50 nM of the E gene target, whereas MES buffer optimization was conducted using 10 nM. After the reaction, significant purple signals were observed in the TL and CL. Finally, the images were recorded and analyzed using ImageJ software.

Optimization of chemicals for Ag staining amplification

After hybridization and Au deposition, the TL and CL showed purple-colored lines. Ag staining was produced by layer-by-layer lamination. Specifically, the glass-fiber papers were treated with different concentrations of $AgNO_3$ solution (0.03%, 0.15%, 0.3% and 3%) and hydroquinone solution (0.3%, 1.5%, 3% and 6%) and dried at 37°C for 30 minutes using 50 nM of the E gene target. After the Ag staining method, the TL and CL displayed distinctly dark black bands. All images were collected and analyzed using Image J software. The excess unreacted hydroquinone and Ag^+ waste remaining after the assay can be treated through biodegradation[4] or chemical methods,[5] or alternatively, collected and

transferred to licensed waste management facilities for proper disposal.

Evaluation of NALFA platform sensitivity

The detection performance of the NALFA platform was evaluated by testing the sensitivity and limit of detection (LOD), using the SARS-CoV-2 E gene as a standard. Briefly, 300 µL of running buffer (1% BSA solution in 1× PBST) containing different concentrations of E gene (0, 0.1, 0.5, 1, 5, 10, 50, 100, 500 and 1000 nM) in 2 × SSC buffer was individually dropped onto the sample pad of the platform. The results were visually observed with the naked eye for qualitative analysis. In addition, the dark signal bands were recorded by a smartphone and analyzed with Image J software. All experiments were performed in triplicate under the same conditions.

Analysis of SARS-CoV-2 Samples

To demonstrate the applicability of the device in clinical settings, we used the sample derived from Vero E6 cells infected with the SARS-CoV-2 Omicron BA.1 viral strain. Clinical samples were collected from critically ill patients admitted to National Taiwan University Hospital, Taipei, Taiwan. The viral collecting procedures used were authorized by the Institutional Review Board of National Taiwan University Hospital (202305113DINB) and were within the institutional guidelines and regulations. The virus-containing liquid was collected after the obvious cytopathic effect (CPE) in the process of culturing the virus, and the extracted RNA was reverse transcribed into cDNA using the SuperScript™ III first-strand synthesis superMix. The reaction mixture comprising 4.0 µL extracted RNA, 1.0 µL Primer, 1.0 µL annealing buffer, and 2.0 µL nuclease-free water was incubated at 65 °C for 5 min. Subsequently, the reaction solution was added to 10.0 µL 2×First-Strand Reaction Mix and 2.0 µL Enzyme Mix at 4 °C, followed by incubation at 25°C for 10 min to allow for annealing. The RNA was then reverse transcribed into cDNA at 50°C for 50 min and the reaction was terminated at 85°C for 5 min. Sequentially, we used PCR to obtain the target sequence. Briefly, each 20 µL reaction mixture consisting of 1.0 µL of cDNA, 10.0 µL KAPA, 0.3 µL SARS-CoV-2 E-forward primer (10.0 µM), 0.3 µL SARS-CoV-2 E-reverse primer (10.0 µM) and 8.4 µL Nuclease-free water were incubated at 95 °C for 3 min, followed by 30 cycles of 95°C for 3 s and 60°C for 20 s. Before testing, the 20.0 µL product was denatured by treating it for 10 min, then mixed with running buffer in 2× SSC buffer, and dropped onto the sample pad of the platform. Following dual-signal amplification, images were recorded and analyzed using smartphone and Image J software.

Table S1. Oligonucleotides sequences

Name	Sequence (5' - 3')
SARS-CoV-2 E gene	GCTAGTTACACTAGCCATCCTTACTGCGCTTCGATTGTGTGCGTACTGCT
polyA DNA	GGATGGCTAGTGTAACTAGCCCCCCCCCCCAAAAAAAAAAAAAAAAAAAA
Biotinylated capture DNA	Biotin-CCCCCCAGCAGTACGCACACAATCGA
Biotinylated control DNA	GCTAGTTACACTAGCCATCCAAAAAA-Biotin
SARS-CoV-2 E-forward primer	GCTAGTTACACTAGCCATCC
SARS-CoV-2 E-reverse primer	AGCAGTACGCACACAATCGA

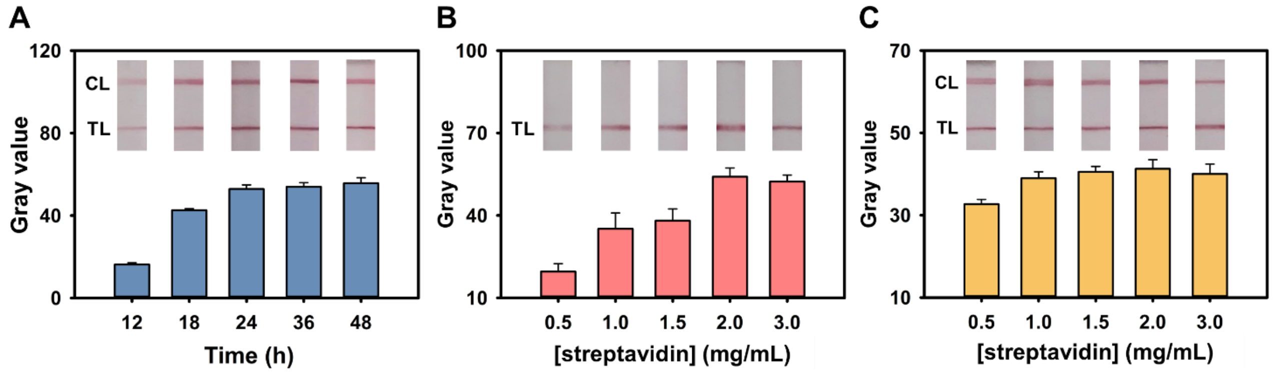

Figure S1. (A) Optimized incubation time for polyA DNA-AuNPs synthesis. (B) Different concentrations of streptavidin binding biotinylated capture DNA on the TL. (C) Different concentrations of streptavidin binding biotinylated control DNA on the CL. All images were collected and analyzed using Image J software, and the experiments were performed in triplicate under the same conditions.

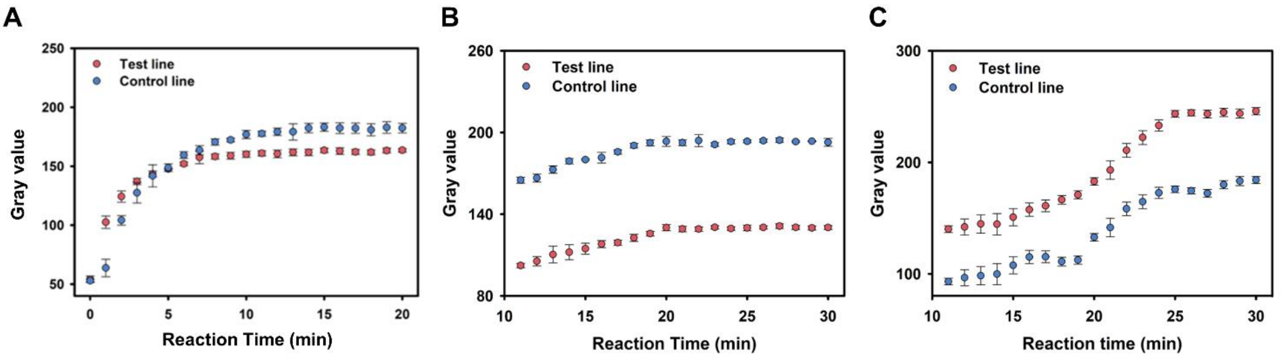

Figure S2. (A)Time-dependent using the SARS-CoV-2 E-gene as standard in NALFA testing recorded with a smartphone. (B) Time-dependent amplification using Au deposition on the NALFA platform. (C) Time-dependent amplification using Au deposition and Ag staining on the NALFA platform in one-step operation. (n=3)

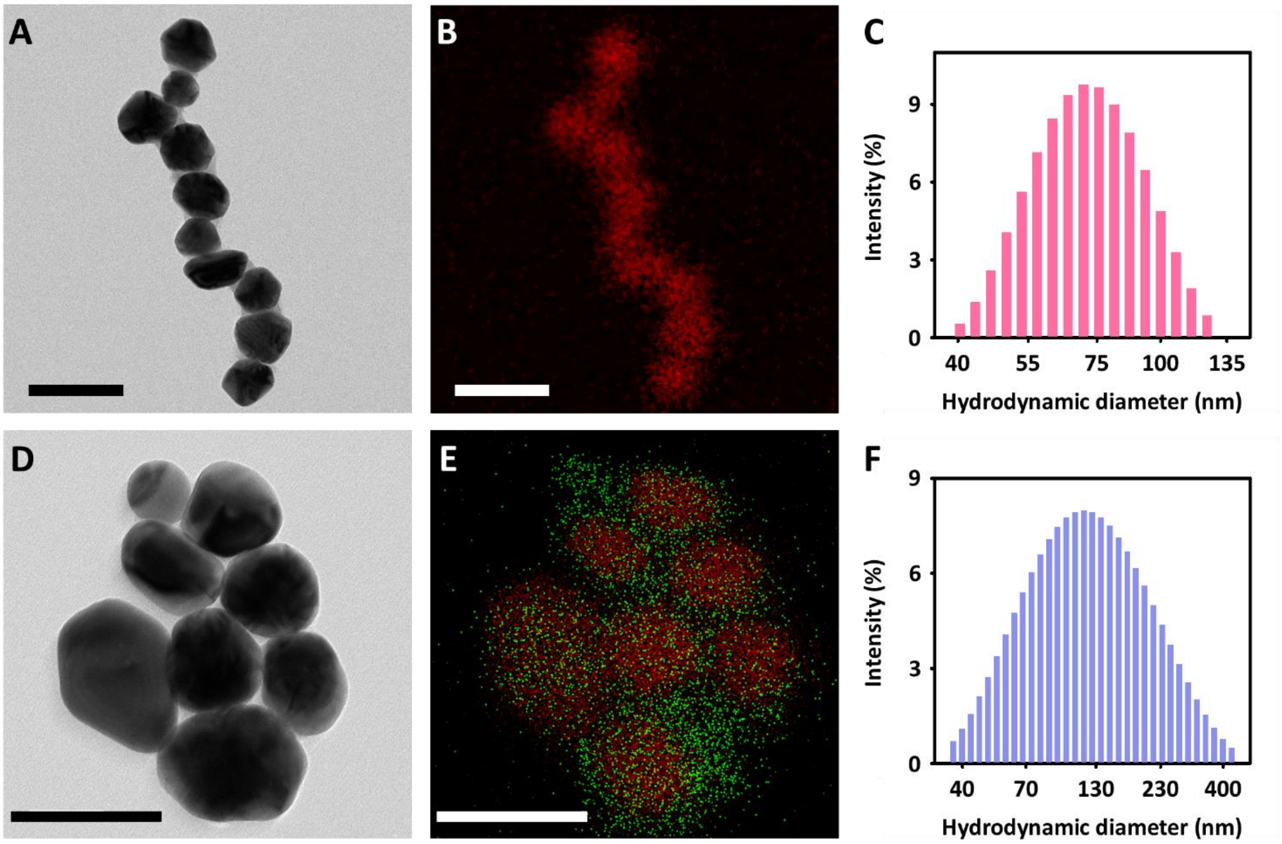

Figure S3. Characterization of the amplification structures. (A) STEM image, (B) Au elemental mapping, and (C) hydrodynamic diameter analysis of the Au deposition amplification. (D) STEM image, (E) elemental mapping of Au (red) and Ag (green), and (F) hydrodynamic diameter analysis of the Au deposition with Ag staining. (Scale bar = 100 nm).

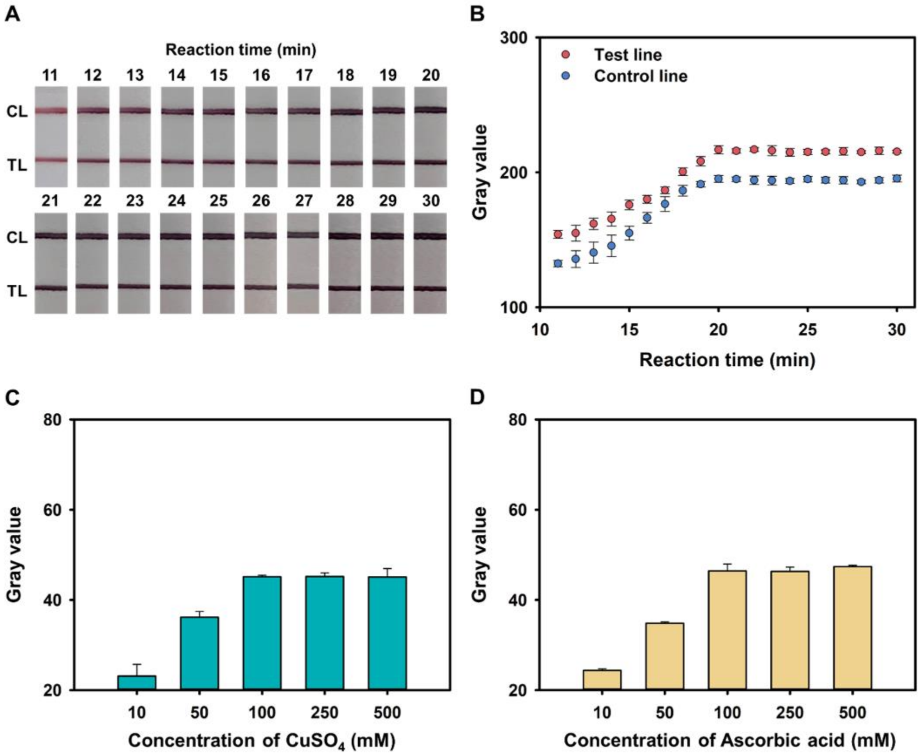

Figure S4. (A) Time-dependent amplification using copper deposition on the NALFA platform. (B) The images were converted into grayscale with Image J software and analyzed from the NALFA platform. Optimization of (C) $CuSO_4$ and (D) L-Ascorbic acid concentrations for signal amplification. All images of the NALFA platform were recorded and analyzed using grayscale with Image J software. All experiments were performed in triplicate under the same conditions.

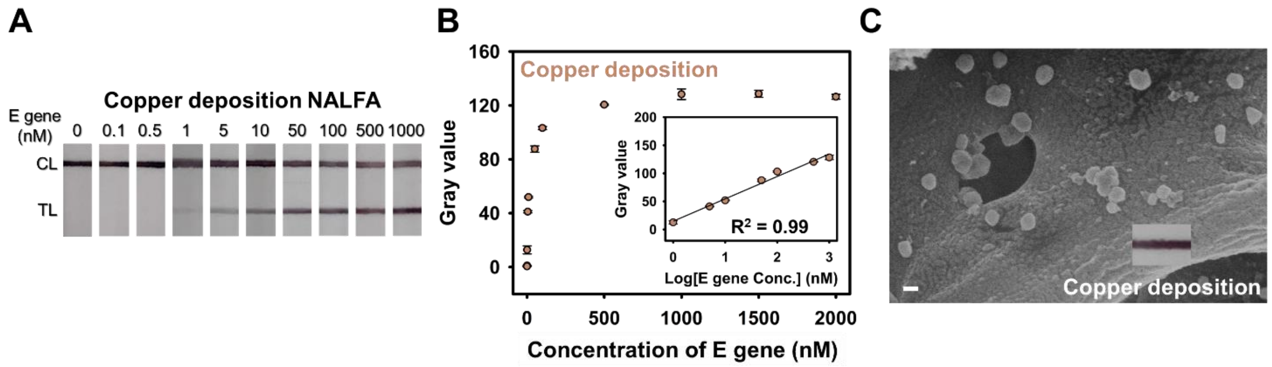

Figure S5. (A) The images after using Cu deposition amplification based on different concentrations of SARS-CoV-2 E gene standards and (B) the titration and calibration curve determined by analysis of grayscale images. (C) The SEM image of the TL was recorded after using Cu deposition. The scale bar is 100 nm.

Table S2. Recovery experiment of Omicron BA.1 samples.

**BA. 1 concentration (copies/μL)**	**Detected concentration (copies/μL)**	**Recovery (%)**
3×10^{8}	3.32×10^{8}	111
6×10^{8}	6.52×10^{8}	108
3×10^{9}	3.28×10^{9}	109
6×10^{9}	6.35×10^{9}	106
3×10^{10}	3.06×10^{10}	102
6×10^{10}	6.12×10^{10}	102
3×10^{11}	2.94×10^{11}	98.1
6×10^{11}	5.73×10^{11}	95.4

Table S3. Comparison of analytical performance, cost, detection time, and operational simplicity among five representative SARS-CoV-2 LFA-based detection methods.

Methods	LOD	cost	time	Ease of operation	reference
RT-RAA + NALFA	★★☆	★★★	★★★	★★★	6
RPA–TeaPNA–LFA	★★☆	★★☆	★★☆	★★☆	7
RT-LAMP + LFA	★★☆	★★☆	★★★	★★★	8
Plasmonic Fluor–LFA	★★★	★★☆	★★☆	★★☆	9
CRISPR/Cas13a + RPA + LFA	★★★	★☆☆	★★☆	★☆☆	10
This work	★★☆	★★★	★★★	★★★	

REFERENCES

(1) Hayat, M. A. *Colloidal gold: principles, methods, and applications*; Elsevier, 2012.

(2) Pei, H.; Li, F.; Wan, Y.; Wei, M.; Liu, H.; Su, Y.; Chen, N.; Huang, Q.; Fan, C. Designed diblock oligonucleotide for the synthesis of spatially isolated and highly hybridizable functionalization of DNA–gold nanoparticle nanoconjugates. *Journal of the American Chemical Society* **2012**, *134* (29), 11876-11879.

(3) Mao, M.; Xie, Z.; Ma, P.; Peng, C.; Wang, Z.; Wei, X.; Liu, G. Design and optimizing gold nanoparticle-cDNA nanoprobes for aptamer-based lateral flow assay: Application to rapid detection of acetamiprid. *Biosensors and Bioelectronics* **2022**, *207*, 114114.

(4) Enguita, F. J.; Leitão, A. L. Hydroquinone: environmental pollution, toxicity, and microbial answers. *BioMed research international* **2013**, *2013* (1), 542168. Sun, H.; Tai, X.; Chen, Y.; Gao, T.; Zhang, W.; Liu, G.; Chen, X.; Dyson, P. Efficient degradation of hydroquinone by a metabolically engineered Pseudarthrobacter sulfonivorans strain. *Archives of Microbiology* **2022**, *204* (9), 588.

(5) Garete, A. J.; Menor, T. L.; Mena, M. *Eco-Friendly Method of Silver Recovery from Small Scale Mining Waste Solutions*; 2019.

(6) Vindeirinho, J. M.; Pinho, E.; Gomes, L.; Guiomar, R.; Kobialka, R. M.; Abd El Wahed, A.; Azevedo, N. F.; Almeida, C. RT-RAA with a lateral flow assay readout based on ssDNA hybridization for detection of RNA viruses–the case of SARS-CoV-2. *Sensors and Actuators B: Chemical* **2025**, *426*, 136864.

(7) Gupta, R.; Gupta, P.; Wang, S.; Melnykov, A.; Jiang, Q.; Seth, A.; Wang, Z.; Morrissey, J. J.; George, I.; Gandra, S. Ultrasensitive lateral-flow assays via plasmonically active antibody-conjugated fluorescent nanoparticles. *Nature Biomedical Engineering* **2023**, *7* (12), 1556-1570.

(8) Zheng, T.; Li, X.; Si, Y.; Wang, M.; Zhou, Y.; Yang, Y.; Liang, N.; Ying, B.; Wu, P. Specific lateral flow detection of isothermal nucleic acid amplicons for accurate point-of-care testing. *Biosensors and Bioelectronics* **2023**, *222*, 114989.

(9) Cao, G.; Huo, D.; Chen, X.; Wang, X.; Zhou, S.; Zhao, S.; Luo, X.; Hou, C. Automated, portable, and high-throughput fluorescence analyzer (APHF-analyzer) and lateral flow strip based on CRISPR/Cas13a for sensitive and visual detection of SARS-CoV-2. *Talanta* **2022**, *248*, 123594.

(10) Ge, A.; Liu, F.; Teng, X.; Cui, C.; Wu, F.; Liu, W.; Liu, Y.; Chen, X.; Xu, J.; Ma, B. A Palm Germ-Radar (PaGeR) for rapid and simple COVID-19 detection by reverse transcription loop-mediated isothermal amplification (RT-LAMP). *Biosensors and Bioelectronics* **2022**, *200*, 113925.
